# Bright’s Disease

**Published:** 1888-10

**Authors:** 


### Bright's Disease.

John Bright was born in 1811. He made a tour of the Holy Land at the age of twenty-four, but did not desire to purchase it, owing to the existence to a flaw in the title. On his return from the Orient he discovered that what was most needed both in Europe and America was a good reliable disease for the better classes. The poor and humble were well supplied, but the rich, the aristocratic and patrician statesmen, corned heads and porkists of the two lands, languished for a good reliable disease that poor people could not obtain. So he began to sit up nights and perfect Bright's Disease. He gained the prize at the Paris Exposition, and honorable mention at the great Centennial celebration at Philadelphia, for "meritorious and effective disease for the better classes." Since that time he has been gratified to notice that the very best people, both in his own land and in this, are handling Bright's disease. It has been kept out of the reach of the poor, and to die from this ailment has been regarded as a proud distinction.—*Bill Nye's Biography of Noted Men.*

